# Milk Consumption and Mortality from All Causes, Cardiovascular Disease, and Cancer: A Systematic Review and Meta-Analysis

**DOI:** 10.3390/nu7095363

**Published:** 2015-09-11

**Authors:** Susanna C. Larsson, Alessio Crippa, Nicola Orsini, Alicja Wolk, Karl Michaëlsson

**Affiliations:** 1 Unit of Nutritional Epidemiology, Institute of Environmental Medicine, Karolinska Institutet, SE-171 77 Stockholm, Sweden; E-Mails: alessio.crippa@ki.se (A.C.); nicola.orsini@ki.se (N.O.); alicja.wolk@ki.se (A.W.); 2 Unit of Biostatistics, Institute of Environmental Medicine, Karolinska Institutet, SE-171 77 Stockholm, Sweden; 3 Department of Surgical Sciences, Uppsala University, SE-751 85 Uppsala, Sweden; E-Mail: karl.michaelsson@surgsci.uu.se

**Keywords:** cancer, cardiovascular disease, meta-analysis, milk, mortality

## Abstract

Results from epidemiological studies of milk consumption and mortality are inconsistent. We conducted a systematic review and meta-analysis of prospective studies assessing the association of non-fermented and fermented milk consumption with mortality from all causes, cardiovascular disease, and cancer. PubMed was searched until August 2015. A two-stage, random-effects, dose-response meta-analysis was used to combine study-specific results. Heterogeneity among studies was assessed with the *I*^2^ statistic. During follow-up periods ranging from 4.1 to 25 years, 70,743 deaths occurred among 367,505 participants. The range of non-fermented and fermented milk consumption and the shape of the associations between milk consumption and mortality differed considerably between studies. There was substantial heterogeneity among studies of non-fermented milk consumption in relation to mortality from all causes (12 studies; *I*^2^ = 94%), cardiovascular disease (five studies; *I*^2^ = 93%), and cancer (four studies; *I*^2^ = 75%) as well as among studies of fermented milk consumption and all-cause mortality (seven studies; *I*^2^ = 88%). Thus, estimating pooled hazard ratios was not appropriate. Heterogeneity among studies was observed in most subgroups defined by sex, country, and study quality. In conclusion, we observed no consistent association between milk consumption and all-cause or cause-specific mortality.

## 1. Introduction

Milk is a widely consumed dairy product. Being rich in protein, saturated fat (whole milk), lactose, calcium, and other essential nutrients, milk consumption may influence the risk of disease and mortality. Evidence indicates that milk consumption may be associated with an increased risk of prostate cancer [1] but with a reduced risk of colorectal cancer [2]. Milk consumption has been inconsistently associated with cardiovascular disease [3,4,5] and type 2 diabetes [6,7], and does not appear to reduce the risk of hip fractures [8]. Whether milk consumption is related to all-cause mortality remains unclear. We therefore conducted a systematic review and meta-analysis to evaluate any potential association between non-fermented milk consumption and mortality from all causes, overall cardiovascular disease, and overall cancer. In addition, we assessed whether consumption of fermented milk, which might have antioxidant and anti-inflammatory effects [9,10], is associated with all-cause mortality.

## 2. Experimental Section 

### 2.1. Literature Search

We followed standard criteria for performing and reporting of meta-analyses of observational studies [11]. Studies were identified by a systematic review of the literature until August 2015 by using the electronic PubMed database. No restrictions were imposed. We used the search terms: (dairy OR milk OR yogurt) AND (mortality or death) AND (cohort OR prospective). In addition, we manually searched the reference lists of recent reviews and other retrieved publications to search for further articles. 

### 2.2. Study Selection

We included prospective studies that provided hazard ratios (HRs) with 95% confidence intervals (CI) for at least three categories (including the reference group) of milk consumption in relation to mortality from all causes, overall cardiovascular disease, or overall cancer, We omitted studies that only reported results for total milk products or combined non-fermented and fermented milk because non-fermented and fermented milk may have different associations with mortality.

### 2.3. Data Extraction and Quality Assessment

From each publication, we extracted the first author’s last name, year of publication, name of the cohort, country, sex, age range of the study population, sample size, number of deaths, duration of follow-up, variables adjusted for in the statistical analysis, and HRs with 95% CIs for each category of milk consumption. We extracted the HRs from the most fully adjusted model, except when adjustments were made for major components of milk, such as dietary calcium. Data were extracted separately for women and men if sex-specific results were provided. Study quality was assessed using the Newcastle-Ottawa Scale [12]. The score ranged from 0–9 stars (9 representing the highest quality).

### 2.4. Statistical Analysis

A two-stage, random-effects, dose-response meta-analysis [13,14] was conducted to assess potential nonlinear associations between milk consumption and mortality. This was done by modeling milk consumption by using restricted cubic splines with three knots at fixed percentiles [14]. First, a restricted cubic spline model with two spline transformations was fitted, taking into account the correlation within each set of published relative risks [13,14]. Second, the two regression coefficients and the variance/covariance matrices estimated for each study were combined using a multivariate random-effects meta-analysis [15]. An overall *p-*value was computed by testing that the two regression coefficients were equal to zero. We calculated a *p-*value for nonlinearity by testing that the coefficient of the second spline was equal to zero [16]. The dose-response meta-analysis method requires that (1) risk estimates with CIs are available for at least three exposure categories (including the reference group); (2) the number of cases and participants (or person-time) for each category are known (to be able to estimate variance/covariance matrices); and (3) the mean or median milk consumption for each exposure category is reported in the article or can be estimated.

Heterogeneity among studies was evaluated using the *I*^2^ statistics [17]. Low, moderate-to-high, and substantial heterogeneity was defined by *I*^2^-values of <25%, 25%–75%, and >75%, respectively. To investigate the influence of single studies on the overall results, we conducted a sensitivity analysis in which one study at a time was removed and the rest analyzed. Potential sources of heterogeneity due to sex, country, and study quality were assessed using stratified analysis. The statistical analyses were conducted using the dosresmeta [18] and metaphor [19] packages in R (R Foundation for Statistical Computing, Vienna, Austria) [20]. *p*-values < 0.05 were considered statistically significant.

## 3. Results

### 3.1. Literature Search

**Figure 1:**
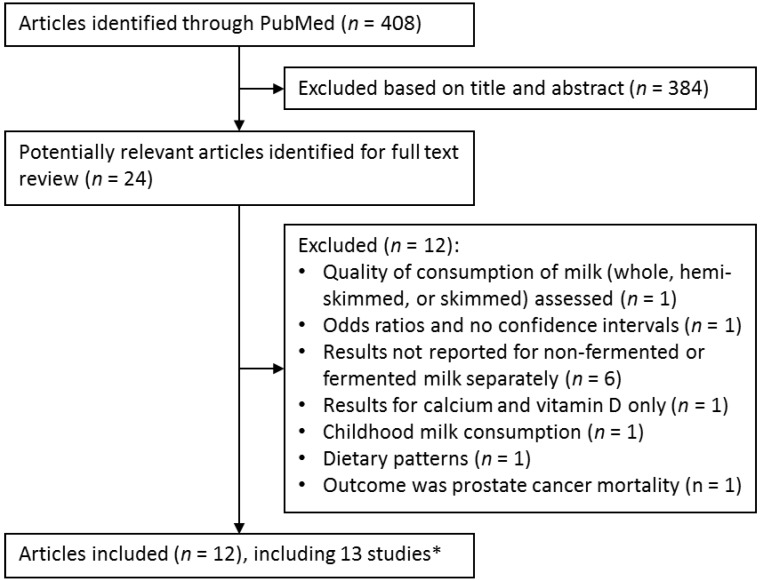
Flow diagram of literature search and study selection. Studies excluded based on title and abstract included experimental studies in animals and *in vitro*, review articles, and other studies unrelated to milk consumption and mortality. * One article reported results from two separate cohorts and one article reported results for fermented milk only.

We identified 12 prospective studies [21,22,23,24,25,26,27,28,29,30,31] (one article presented results from two separate cohort studies [30]) that reported HRs of mortality from all causes (*n* = 12), cardiovascular disease (*n* = 5), or cancer (*n* = 4) in relation to non-fermented milk consumption (Figure 1). Six of those studies (five articles) also provided results on fermented milk (yogurt and/or soured milk) [25,26,27,28,30] consumption. We identified an additional study on consumption of yogurt in relation to mortality [32].

### 3.2. Study Characteristics

Characteristics of the included studies on non-fermented milk consumption in relation to all-cause mortality are shown in Table 1. Four studies were conducted in the UK or Scotland, two in Sweden, two in the US, and one each in the Netherlands, Japan, and Australia. One study included cohorts from 10 European countries. Combined, these 12 studies included 70,743 deaths among 367,505 participants. All studies controlled for age and sex (if applicable). Most studies also adjusted for smoking (*n* = 11), body mass index (*n* = 10), alcohol consumption or drinking status (*n* = 9), total energy intake (*n* = 8), physical activity (*n* = 7), and markers of socioeconomic status (*n* = 7). Few studies adjusted for other food items or a healthy eating pattern (*n* = 5). Information on milk consumption was obtained through self-report in all studies. Table S1 presents the scores assigned to each study and Figure S1 lists details of how the criteria of study quality were applied.

**Table 1:** Characteristics of studies included in meta-analysis of milk consumption and all-cause mortality.

First Author, Year	Cohort Name	Country	No. of Deaths	Sex (No. of Participants)	Age Range, Years	Duration of Follow-up, Years	Milk Intake Categories	HR (95% CI)	Adjustments
Mann, 1997 [21]	NA	UK	392	Women and men (10,802)	16–79	13.3	<280 mL/day ^a^	1.00 (ref.)	Age, sex, smoking, and social class
							280 mL/day	0.70 (0.55–0.88)	
							>280 mL/day	0.87 (0.68–1.13)	
Ness, 2001 [22]	Collaborative Study	Scotland	2350	Men (5,765)	35–64	25	<190 mL/day ^a^	1.00 (ref.)	Age, education, social class, father’s social class, smoking, BMI, diastolic blood pressure, cholesterol, adjusted FEV1, deprivation category, siblings, car user, angina, ECG ischemia, bronchitis, and alcohol intake
							190–750 mL/day	0.90 (0.83–0.97)	
							≥760 mL/day	0.81 (0.61–1.09)	
Elwood, 2004 [23]	Caerphilly Cohort Study	UK	811	Men (2512)	45–59	20–24	0	1.00 (ref.)	Age, social class, smoking, BMI, systolic blood pressure, prior vascular disease, intake of fat, alcohol, and total energy
							<280 mL/day ^a^	0.99 (0.73–1.34)	
							280–570 mL/day	0.98 (0.72–1.35)	
							>570 mL/day	1.20 (0.80–1.80)	
Paganini-Hill, 2007 [24]	Leisure World Cohort Study	US	11,396	Women and men (13,624)	44–101	23	0 glasses/day	1.00 (ref.)	Age, sex, smoking, BMI, exercise, histories of hypertension, angina, heart attack, stroke, diabetes, rheumatoid arthritis, and cancer, alcohol intake
							<1 glasses/day	0.95 (0.90–1.00)	
							1 glasses/day	1.01 (0.96–1.06)	
							≥2 glasses/day	1.04 (0.98–1.10)	
Bonthuis, 2010 [25]	NA	Australia	177	Women and men (1529)	25–78	14.4	<198 g/day	1.00 (ref.)	Age, sex, school leaving age, smoking, BMI, physical activity level, dietary supplement use, beta-carotene treatment during trial, presence of any medical condition, and alcohol and total energy intake
							198–328 g/day	0.85 (0.54–1.33)	
							≥329 g/day	0.93 (0.59–1.48)	
Goldbohm, 2011 [26]	Netherlands Cohort Study	Netherlands	5478 in women: 10,658 in men	Women (62,573) and men (58,279)	55–69	10	Women	Women	Age, education, smoking, BMI, non-occupational and occupational physical activity, multivitamin use, intake of fruits and vegetables, monounsaturated fat, polyunsaturated fat, alcohol, and total energy
							Q1: 0 g/day ^c^	1.00 (ref.)	
							Q2: 21 g/day	0.96 (0.87–1.05)	
							Q3: 52 g/day	0.96 (0.88–1.04)	
							Q4: 107 g/day	0.94 (0.86–1.04)	
							Q5: 238 g/day	1.00 (0.91–1.09)	
							Men	Men	
							Q1: 0 g/day ^c^	1.00 (ref.)	
							Q2: 34 g/day	0.99 (0.93–1.05)	
							Q3: 90 g/day	1.00 (0.94–1.08)	
							Q4: 156 g/day	1.01 (0.94–1.08)	
							Q5: 342 g/day	1.02 (0.95–1.09)	
Soedamah-Muthu, 2013 [27]	Whitehall II prospective cohort study	UK	237	Women and men (4526)	56 ^b^	11.7	147 g/day	1.00 (ref.)	Age, sex, ethnicity, employment grade, smoking, BMI, physical activity, family history of CHD/hypertension, fruit and vegetables, bread, meat, fish, coffee, tea, alcohol, and total energy intake
							294 g/day	0.98 (0.72–1.34)	
							441 g/day (median)	0.89 (0.64–1.25)	
Dik, 2014 [28]	European Prospective Investigation into Cancer and Nutrition	10 European countries ^d^	1525	Women and men (3859)^e^	64.2 ^b^	4.1	<24 g/day	1.00 (ref.)	Age, sex, center, smoking, pre-diagnostic BMI, tumor sub-site (colon and rectum), disease stage, differentiation grade, and total energy intake
							24–147 g/day	1.05 (0.90–1.23)	
							48–293 g/day	1.04 (0.89–1.22)	
							>293 g/day	1.21 (1.03–1.43)	
Yang, 2014 [29]	Cancer Prevention Study II Nutrition Cohort	US	949	Women and men (2284) ^e^	64 ^b^	17	Q1 ^f^	1.00 (ref.)	Age, sex, tumor stage, folate and total energy intake
							Q2	1.01 (0.84–1.23)	
							Q3	0.99 (0.82–1.19)	
							Q4	0.95 (0.79–1.15)	
Michaëlsson, 2014 [30]	Swedish Mammography Cohort	Sweden	15,541	Women (61,433)	39–74	20.1	<200 g/day	1.00 (ref.)	Age, education, living alone, smoking status, BMI, height, physical activity, cortisone use, use of estrogen replacement therapy, nulliparity, Charlson’s comorbidity index, calcium and vitamin D supplementation, healthy dietary pattern, alcohol and total energy intake
							200–399 g/day	1.21 (1.16–1.25)	
							400–599 g/day	1.60 (1.53–1.68)	
							≥600 g/day	1.93 (1.80–2.06)	
Michaëlsson, 2014 [30]	Cohort of Swedish Men	Sweden	10,112	Men (45,339)	45–79	11.2	<200 g/day	1.00 (ref.)	Age, education, living alone, smoking status, BMI, height, physical activity, cortisone use, Charlson’s comorbidity index, calcium and vitamin D supplementation, healthy dietary pattern, alcohol and total energy intake
							200–399 g/day	0.99 (0.94–1.05)	
							400–599 g/day	1.05 (1.00–1.11)	
							≥600 g/day	1.10 (1.03–1.17)	
Wang, 2015 [31]	Japan Collaborative Cohort Study	Japan	9572 in women; 12,203 in men	Women (55,341); Men (39,639)	40–79	19	Women	Women	Age, education, smoking status, drinking status, BMI, physical activity, sleeping duration, participation in health check-ups, history of hypertension, diabetes, and liver disease, green-leafy vegetable intake
							Never	1.00 (ref.)	
							1–2 times/month	1.00 (0.91–1.05)	
							1–2 times/week	0.98 (0.91–1.05)	
							3–4 times/week	0.91 (0.85–0.98)	
							Almost daily	0.96 (0.91–1.01)	
							Men	Men	
							Never	1.00 (ref.)	
							1–2 times/month	0.92 (0.86–0.99)	
							1–2 times/week	0.91 (0.85–0.96)	
							3–4 times/week	0.89 (0.84–0.96)	
							Almost daily	0.93 (0.89–0.98)	

Abbreviations: BMI, body mass index; CHD, coronary heart disease; CI, confidence interval; ECG, electrocardiogram; FEV1, forced expiratory volume in the first second. HR, hazard ratio; NA, not available; Q, quartile or quintile. ^a^ Amount was expressed in pints (1 pint = 568 mL). ^b^ Mean age. ^c^ Median intake in each tertile. ^d^ Including Denmark, France, Germany, Greece, Italy, Netherlands, Norway, Spain, Sweden, and UK. ^e^ Colorectal cancer patients. ^f^ Quartiles for women were 0, 0.1–5.0, 5.1–10.0, and ≥10.1 serving/week; quartiles for men were 0, 0.1–5.6, 5.7–10.4, and ≥10.5 serving/week. One serving was assumed to equal 200 mL.

### 3.3. Non-Fermented Milk

**Figure 2:**
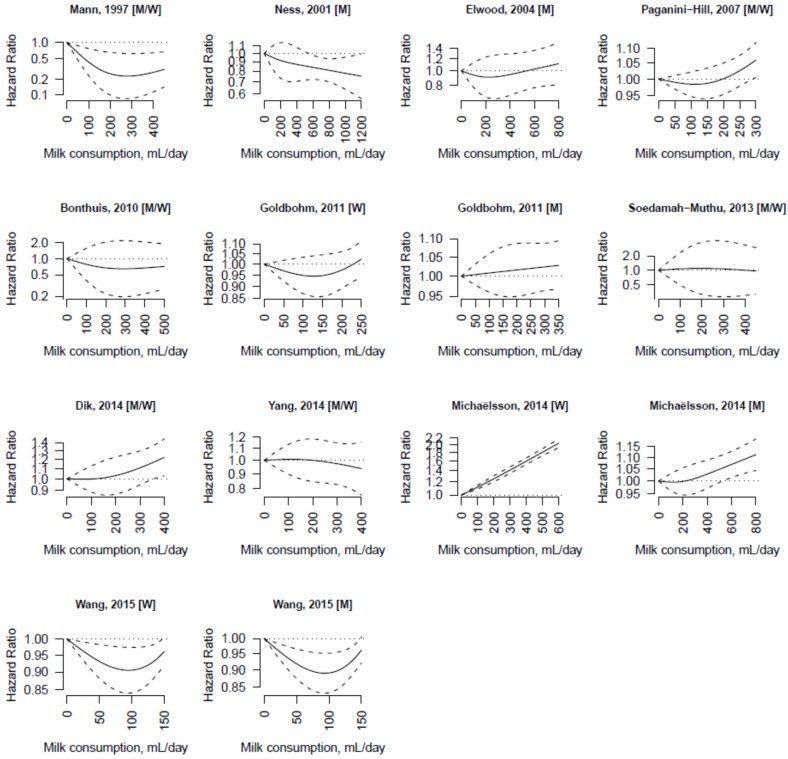
Dose-response association between non-fermented milk consumption and all-cause mortality in individual studies. The hazard ratios are plotted on a log scale.

The range of non-fermented milk consumption and the shape of the association between milk consumption and all-cause mortality differed between studies (Figure 2). Due to substantial heterogeneity among studies (*I*^2^ = 94%), estimating a pooled HR was not appropriate. In a sensitivity analysis, in which one study at the time was removed and the rest analyzed to assess the influence of single studies on the overall results, we found that the Swedish Mammography Cohort [30] contributed most to the heterogeneity. After excluding this study, the heterogeneity was reduced (*I*^2^ = 58%). Heterogeneity among studies was observed in most subgroups defined by sex (women: *I*^2^ = 93.9%; men *I*^2^ = 70%; both: *I*^2^ = 47%), country (UK/Scotland: *I*^2^ = 44%; Sweden: *I*^2^ = 99%; rest of Europe: *I*^2^ = 19%; US: *I*^2^ = 40%), and study quality (Newcastle-Ottawa Scale < 7: *I*^2^ = 45%; ≥ 7: *I*^2^ = 97%).

**Figure 3:**
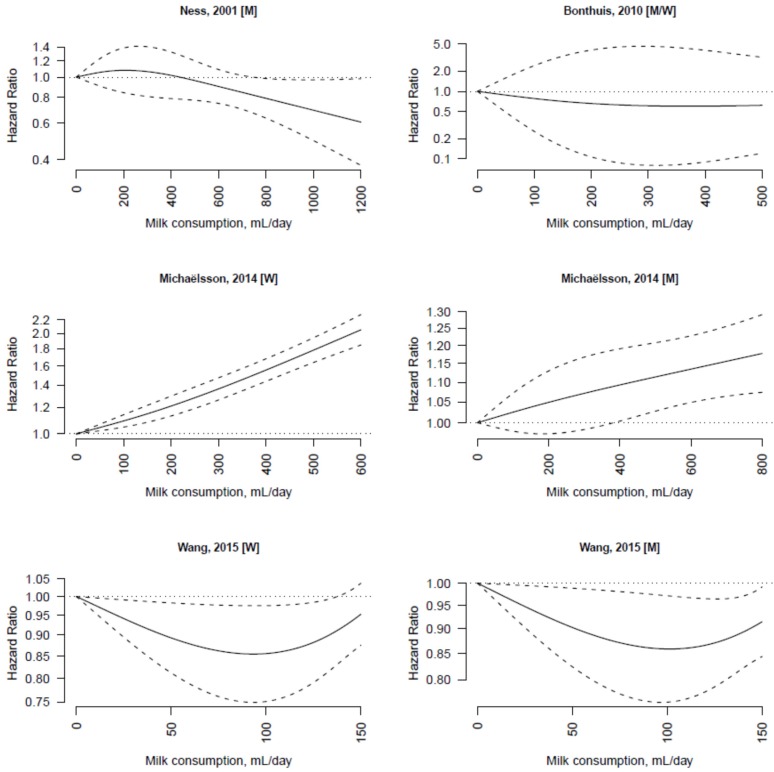
Dose-response association between non-fermented milk consumption and cardiovascular disease mortality in individual studies. The hazard ratios are plotted on a log scale.

**Figure 4:**
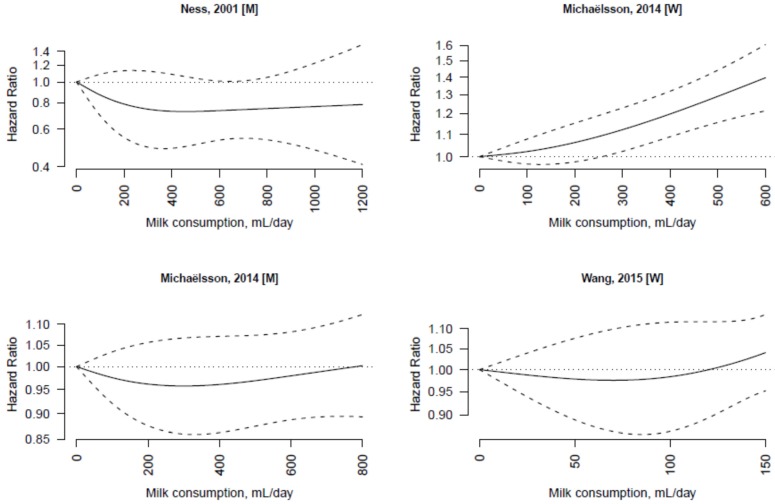

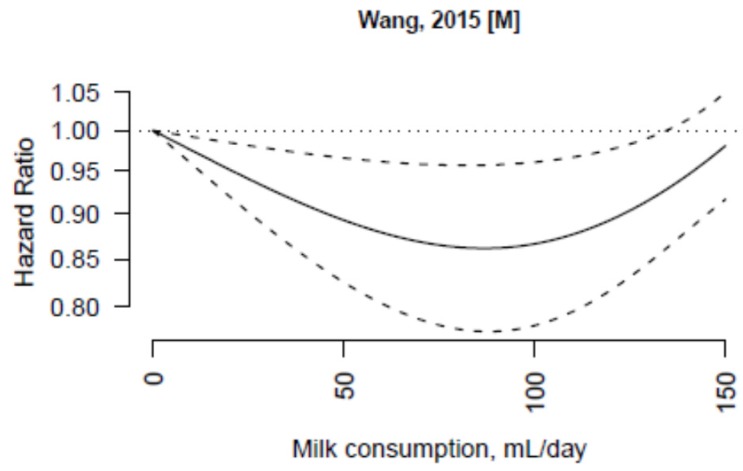
Dose-response association between non-fermented milk consumption and cancer mortality in individual studies. The hazard ratios are plotted on a log scale.

The dose-response associations of milk consumption with cardiovascular disease and cancer mortality in individual studies are shown in Figure 3 and Figure 4, respectively. There was substantial heterogeneity among studies of cardiovascular disease (*I*^2^ = 93%) and cancer (*I*^2^ = 75%) mortality.

### 3.4. Fermented Milk

The HRs of all-cause mortality by levels of fermented milk consumption are presented in Table S2. Most studies indicated a U-shaped association between fermented milk consumption and all-cause mortality (Figure S2). The range of fermented milk consumption differed among studies and there was substantial heterogeneity among studies (*I*^2^ = 88%).

## 4. Discussion

This systematic review and meta-analysis found substantial heterogeneity among studies of non-fermented and fermented milk consumption and mortality from all causes, cardiovascular disease, and cancer. Due to the large variation in the range of milk consumption across populations and the considerable heterogeneity, it was not appropriate to pool the results.

Among the 12 studies of non-fermented milk consumption, Michaëlsson *et al.* [30] observed statistically significant positive associations of non-fermented milk consumption with all-cause and cardiovascular disease mortality in cohorts of Swedish women and men. In the same study [30], non-fermented milk consumption was also positively associated with cancer mortality in the female cohort (but the association was weaker than for all-cause and cardiovascular disease mortality) but not in the male cohort. Likewise, Dik *et al.* [28] observed a positive association between high consumption of non-fermented milk and all-cause mortality in a pooled analysis of cohort studies from 10 European countries. In contrast, Mann *et al.* [21] and Ness *et al.* [22] found some indication of an inverse relation between non-fermented milk consumption and all-cause mortality. The study by Mann *et al.* [21] did not control for potential confounders such as body mass index, physical activity, alcohol consumption, and diet. Wang *et al.* [31] observed a U-shaped association of non-fermented milk consumption with all-cause and cardiovascular disease mortality in a population of Japanese adults with very low milk consumption. The other six studies reported no significant relation between non-fermented milk consumption and all-cause [23,24,25,26,27,29] or cardiovascular disease [25] mortality. A potential explanation for the inconsistent findings may be related to the different range of milk consumption in different populations. Milk consumption was high and the range of consumption was large in the studies by Michaëlsson *et al.* [30]. Although there was a wide range of milk consumption also in the studies by Ness *et al.* [22] and Elwood *et al.* [23], those studies had limited power to detect a statistically significant association because of a small number of deaths and participants in the highest exposure category. Furthermore, it was not totally clear if the reported milk consumption in those two studies included non-fermented milk only or also fermented milk.

In addition to the different range of milk consumption, the proportion of different types of milk (e.g., whole milk, reduced-fat and fat-free milk, organic milk, and lactose-free milk) consumed is likely to vary and could contribute to the disparate findings. Moreover, the composition of milk may differ. For example, the proportion of conjugated linoleic acid in milk fat depends on what the cows are fed [33]. Studies with animal models have shown that the predominant conjugated linoleic acid isomer (cis-9,trans-11) has anti-carcinogenic and anti-atherogenic activities [33].

The confounders controlled for in the included studies differed, and this may also, in part, explain the inconsistent results. Whereas most studies adjusted for major risk factors for mortality (e.g., age, sex, smoking, body mass index, physical activity, and alcohol consumption), few studies controlled for other foods [26,27,31] or a healthy food pattern [30]. Potential dietary confounders include fruits and vegetables [34], red meat and processed meat [35], and coffee [36] which have been associated with all-cause mortality. Most studies had a long follow-up (usually between 10 and 25 years) and, with the exception of the study in women by Michaëlsson *et al.* [30], did not update information on milk consumption during follow-up. This along with the use of a dietary questionnaire to assess milk consumption would most likely have resulted in some misclassification and attenuated HRs. In fact, in the study of Swedish women by Michaëlsson *et al.* [30], the HRs were attenuated when only a single exposure assessment was applied and were similar to the HRs obtained in the Swedish male cohort, which was based on a single assessment of diet.

Two previous meta-analyses have examined the association between total milk consumption (non-fermented and fermented milk/yogurt combined) and all-cause mortality. One of the meta-analyses included eight prospective studies and showed a HR of all-cause mortality of 0.99 (95% CI 0.95–1.03) per 200-g/day increment of total milk consumption [37], but a meta-regression analytical approach to detect non-linear patterns in risk was not applied. In the other meta-analysis, based on five prospective studies, the HR of all-cause mortality was 1.01 (95% CI 0.92–1.11) for the highest *versus* lowest category of total milk consumption [38]. Five of the studies included in one or both of those meta-analyses were excluded from the current meta-analysis because results were only presented for total milk products [39], for milk and yogurt combined [40], or for comparisons of types of milk (skimmed and semi-skimmed *versus* whole milk) [41], results were unpublished [42], or odds ratios without CIs were reported [43]. Among the excluded studies, Fortes *et al.* [40] observed an inverse association between combined milk and yogurt consumption and all-cause mortality (HR = 0.38; 95% CI, 0.14–1.01, for ≥3 times/week *versus* <1 time/week) in a cohort of 162 Italians, whereas Knoops *et al.* [39] reported a positive association between total milk products and all-cause mortality (HR = 1.10; 95% CI, 1.00–1.21, for consumption above *versus* below the median) in a cohort of 3117 elderly adults from 10 European countries. No association was observed in the other three studies [41,42,43].

## 5. Conclusions

In summary, we observed no consistent association between non-fermented or fermented milk consumption and mortality. Further large prospective studies assessing the relation between milk consumption and mortality are warranted.

## Supplementary Files

Supplementary File 1
