# Supplementary material for: Milk Consumption and Mortality from All Causes, Cardiovascular Disease, and Cancer: A Systematic Review and Meta-Analysis
Source: Nutrients. 2015 Sep 11;7(9):7749–63. doi: 10.3390/nu7095363 (PMC4586558; doi:10.3390/nu7095363)
Supplement: Supplementary File 1 [file nutrients-07-05363-s001.docx]

**Supplementary Information**

**Table S1.** Quality assessment of the 12 studies included in meta-analysis of non-fermented milk consumption and all-cause mortality.

| **First Author, Publication Year** | **Cohort Name** | **Selection** | **Comparability** | **Outcome** | **Score *** |
| --- | --- | --- | --- | --- | --- |
| Mann, 1997 [1] | NA | * | * | ** | 4 |
| Ness, 2001 [2] | Collaborative Study | ** | ** | ** | 6 |
| Elwood, 2004 [3] | Caerphilly Cohort Study | ** | ** | ** | 6 |
| Paganini-Hill, 2007 [4] | Leisure World Cohort Study | ** | ** | *** | 7 |
| Bonthuis, 2010 [5] | NA | * | ** | *** | 6 |
| Goldbohm, 2011 [6] | Netherlands Cohort Study | ** | ** | *** | 7 |
| Soedamah-Muthu, 2013 [7] | Whitehall II prospective  cohort study | * | ** | *** | 6 |
| Dik, 2014 [8] | European Prospective Investigation into Cancer and Nutrition | * | ** | ** | 5 |
| Yang, 2014 [9] | Cancer Prevention Study II Nutrition Cohort | * |  | *** | 4 |
| Michaëlsson, 2014 [10] | Swedish Mammography Cohort | ***† | ** | *** | 8 |
| Michaëlsson, 2014 [10] | Cohort of Swedish Men | ***† | ** | *** | 8 |
| Wang, 2015 [11] | Japan Collaborative Cohort Study | *** | ** | *** | 8 |

* Study quality was assessed by using the Newcastle-Ottawa Scale for assessing cohort studies. A study can be awarded a maximum of 9 stars. Figure S1 shows the details of how the criteria were applied. † Did not exclude prevalent cases of cardiovascular disease at baseline but adjusted for Charlson’s comorbidity index and excluded prevalent cancer cases.

**Table S2.** Hazard ratios of all-cause mortality by categories of fermented milk consumption.

| **First Author, Publication Year** | **Sex** | **Fermented Milk Intake Category *** | **HR (95% CI)** | **Adjustment** |
| --- | --- | --- | --- | --- |
| Bonthuis, 2010 [5] | Women and men | <2 g/day † | 1.00 (ref.) | Age, sex, school leaving age, smoking, BMI, physical activity level, dietary supplement use, beta-carotene treatment during trial, presence of any medical condition, alcohol and energy intake |
|  |  | 3–29 g/day | 0.84 (0.53–1.35) |  |
|  |  | 30–700 g/day | 1.20 (0.79–1.83) |  |
| Goldbohm, 2011 [6] | Women | Full-fat | Full-fat | Age, education, smoking, BMI, non-occupational and occupational physical activity, multivitamin use, alcohol, monounsaturated fat, polyunsaturated fat, fruit, vegetables, and energy intake |
|  |  | 0 g/day † | 1.00 (ref.) |  |
|  |  | 53 g/day | 0.93 (0.87–1.00) |  |
|  |  | Low-fat ‡ | Low-fat ‡ |  |
|  |  | 0 g/day † | 1.00 (ref.) |  |
|  |  | 60 g/day | 0.94 (0.88–1.01) |  |
|  |  | 192 g/day | 1.02 (0.95–1.09) |  |
| Goldbohm, 2011 [6] | Men | Full-fat | Full-fat | Age, education, smoking, BMI, non-occupational and occupational physical activity, multivitamin use, alcohol, monounsaturated fat, polyunsaturated fat, fruit, vegetables, and energy intake |
|  |  | 0 g/day † | 1.00 (ref.) |  |
|  |  | 53 g/day | 0.93 (0.88–0.98) |  |
|  |  | Low-fat ‡ | Low-fat ‡ |  |
|  |  | 0 g/day † | 1.00 (ref.) |  |
|  |  | 21 g/day | 0.92 (0.87–0.97) |  |
|  |  | 146 g/day | 0.97 (0.93–1.03) |  |
| Soedamah-Muthu, 2013 [7] | Women and men | 0 g/day † | 1.00 (ref.) | Age, sex, ethnicity, employment grade, smoking, BMI, physical activity, family history of CHD or hypertension, intake of fruit and vegetables, bread, meat, fish, coffee, tea, alcohol, and total energy |
|  |  | 21 g/day | 0.93 (0.69–1.27) |  |
|  |  | 117 g/day | 1.04 (0.77–1.42) |  |
| Dik, 2014 [8] |  | 0 g/day † | 1.00 (ref.) | Age, sex, centre, smoking, pre-diagnostic BMI, energy intake, tumour sub-site (colon and rectum), disease stage, and differentiation grade |
|  |  | 13 g/day | 1.01 (0.85–1.20) |  |
|  |  | 57 g/day | 1.13 (0.96–1.33) |  |
|  |  | 122 g/day | 1.08 (0.92–1.28) |  |

**Table S2.** *Cont.*

| **First Author, Publication Year** | **Sex** | **Fermented Milk Intake Category *** | **HR (95% CI)** | **Adjustment** |
| --- | --- | --- | --- | --- |
| Michaëlsson, 2014 [10] | Women | <1 g/day | 1.00 (ref.) | Age, education, living alone, smoking status, BMI, height, physical activity, cortisone use, use of oestrogen replacement therapy, nulliparity, Charlson’s comorbidity index, calcium and vitamin D supplementation, healthy dietary pattern, and alcohol and total energy intake |
|  |  | 1–199 g/day | 0.76 (0.73–0.80) |  |
|  |  | 200–399 g/day | 0.84 (0.80–0.89) |  |
|  |  | ≥400 g/day | 0.86 (0.78–0.95) |  |
| Michaëlsson, 2014 [10] | Men | <1 g/day | 1.00 (ref.) | Age, education, living alone, smoking status, BMI, height, physical activity, cortisone use, Charlson’s comorbidity index, calcium and vitamin D supplementation, healthy dietary pattern, and alcohol and total energy intake |
|  |  | 1–199 g/day | 0.88 (0.84–0.93) |  |
|  |  | 200–399 g/day | 0.91 (0.86–0.95) |  |
|  |  | ≥400 g/day | 0.90 (0.85–0.96) |  |
| Praagman, 2015 [12] | Women and men | 3.8 g/day † | 1.00 (ref.) | Age, sex, total energy intake, smoking, BMI, physical activity, education, hypertension at baseline, alcohol, fruit, vegetables, and energy intake |
|  |  | 26.2 g/day | 0.95 (0.85–1.06) |  |
|  |  | 62.9 g/day | 0.94 (0.84–1.06) |  |
|  |  | 144.5 g/day | 0.95 (0.85–1.07) |  |

Abbreviations: BMI, body mass index; CHD, coronary heart disease; CI, confidence interval; HR, hazard ratio. * Median consumption of fermented milk/yogurt in grams per day, either obtained from the article or estimated based on the cut-offs for each exposure category. † Median intake in the category. ‡ Results for low-fat fermented milk consumption were used in the dose-response meta-analysis.


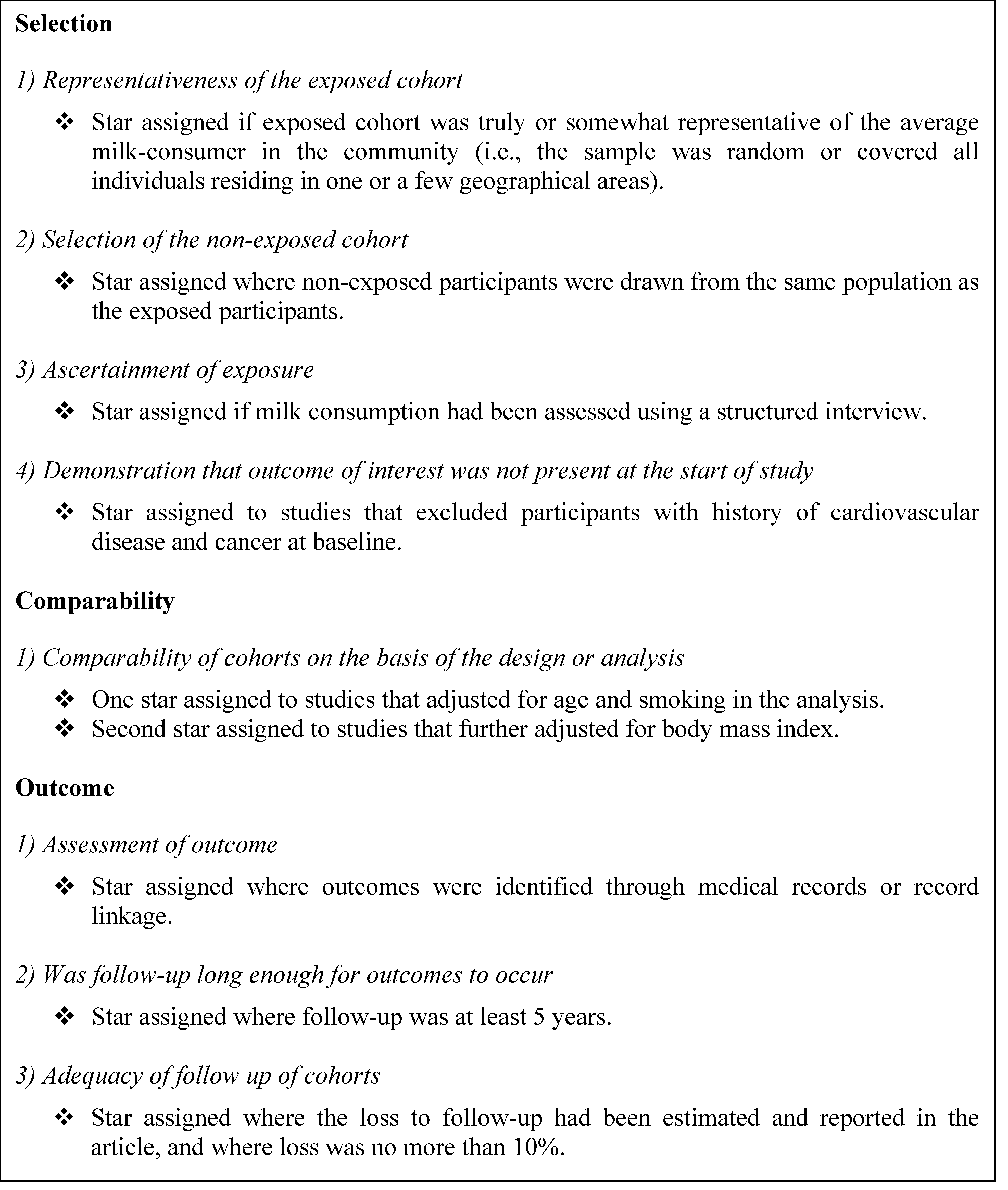


**Figure S1.** Newcastle-Ottawa Scale for Cohort Studies: Details of how the criteria were applied for cohort studies assessing the association between milk consumption and
all-cause mortality.

**Figure S2.** Dose-response association between fermented milk consumption and all-cause mortality in individual studies. The hazard ratios are plotted on a log scale.

**References**

1. Mann, J.I.; Appleby, P.N.; Key, T.J.; Thorogood, M. Dietary determinants of ischaemic heart disease in health conscious individuals. *Heart* **1997**, *78*, 450–455.

2. Ness, A.R.; Smith, G.D.; Hart, C. Milk, coronary heart disease and mortality. *J. Epidemiol. Community Health* **2001**, *55*, 379–382.

3. Elwood, P.C.; Pickering, J.E.; Fehily, A.M.; Hughes, J.; Ness, A.R. Milk drinking, ischaemic heart disease and ischaemic stroke I. Evidence from the Caerphilly cohort. *Eur. J. Clin. Nutr.* **2004**, *58*, 711–717.

4. Paganini-Hill, A.; Kawas, C.H.; Corrada, M.M. Non-alcoholic beverage and caffeine consumption and mortality: The Leisure World Cohort Study. *Prev. Med.* **2007**, *44*, 305–310.

5. Bonthuis, M.; Hughes, M.C.; Ibiebele, T.I.; Green, A.C.; van der Pols, J.C. Dairy consumption and patterns of mortality of Australian adults. *Eur. J. Clin. Nutr.* **2010**, *64*, 569–577.

6. Goldbohm, R.A.; Chorus, A.M.; Galindo Garre, F.; Schouten, L.J.; van den Brandt, P.A. Dairy consumption and 10-y total and cardiovascular mortality: A prospective cohort study in the Netherlands. *Am. J. Clin. Nutr.* **2011**, *93*, 615–627.

7. Soedamah-Muthu, S.S.; Masset, G.; Verberne, L.; Geleijnse, J.M.; Brunner, E.J. Consumption of dairy products and associations with incident diabetes, CHD and mortality in the Whitehall II study. *Br. J. Nutr.* **2013**, *109*, 718–726.

8. Dik, V.K.; Murphy, N.; Siersema, P.D.; Fedirko, V.; Jenab, M.; Kong, S.Y.; Hansen, C.P.; Overvad, K.; Tjonneland, A.; Olsen, A; *et al*. Prediagnostic intake of dairy products and dietary calcium and colorectal cancer survival—Results from the EPIC cohort study. *Cancer Epidemiol. Biomarkers Prev.* **2014**, *23*, 1813–1823.

9. Yang, B.; McCullough, M.L.; Gapstur, S.M.; Jacobs, E.J.; Bostick, R.M.; Fedirko, V.; Flanders, W.D.; Campbell, P.T. Calcium, vitamin D, dairy products, and mortality among colorectal cancer survivors: the Cancer Prevention Study-II Nutrition Cohort. *J. Clin. Oncol.* **2014**, *32*, 2335–2343.

10. Michaëlsson, K.; Wolk, A.; Langenskiold, S.; Basu, S.; Warensjo Lemming, E.; Melhus, H.; Byberg, L. Milk intake and risk of mortality and fractures in women and men: Cohort studies. *BMJ* **2014**, *349*, g6015.

11. Wang, C.; Yatsuya, H.; Tamakoshi, K.; Iso, H.; Tamakoshi, A. Milk drinking and mortality: Findings from the Japan collaborative cohort study. *J. Epidemiol.* **2015**, *25*, 66–73.

12. Praagman, J.; Dalmeijer, G.W.; van der Schouw, Y.T.; Soedamah-Muthu, S.S.; Monique Verschuren, W.M.; Bas Bueno-de-Mesquita, H.; Geleijnse, J.M.; Beulens, J.W. The relationship between fermented food intake and mortality risk in the European Prospective Investigation into Cancer and Nutrition-Netherlands cohort. *Br. J. Nutr.* **2015**, *113*, 498–506.

© 2015 by the authors; licensee MDPI, Basel, Switzerland. This article is an open access article distributed under the terms and conditions of the Creative Commons Attribution license (http://creativecommons.org/licenses/by/4.0/).
